# Assessment of dysfunctional tunneled hemodialysis catheters and outcome of endovascular salvage techniques: a simple solution to a complex problem

**DOI:** 10.3389/fcvm.2023.1063450

**Published:** 2023-08-17

**Authors:** Tao Xu, Ni Zeng, Nan Li

**Affiliations:** ^1^ Department of Interventional Radiology, Shenzhen People’s Hospital, The Second Clinical Medical College, Jinan University, The First Affiliated Hospital, Southern University of Science and Technology; ^2^ Center for Translational Medicine, Institute of Precision Medicine, The First Affiliated Hospital, Sun Yat-sen University, Guangzhou, China; ^3^ Department of Interventional Radiology, Guangzhou First People’s Hospital, Guangzhou, China

**Keywords:** tunneled cuffed catheters, hemodialysis, computed tomography venography, endovascular techniques, central vein stenosis

## Abstract

**Objective:**

The aim of this study was to evaluate the causes of the dysfunctional tunneled cuffed catheters (TCCs) using multi-spiral computed tomography venography (MSCTV), and to analyze the outcomes of endovascular salvage techniques.

**Material and methods:**

This retrospective review data from 27 patients who experienced TCC dysfunction between July 1, 2016 and January 31, 2021 was conducted. Patients’ demographic data, clinical signs and symptoms, and imaging data were collected from interventional radiology database.

**Results:**

MSCTV showed a range of abnormalities in the hemodialysis (HD) patients, including central venous occlusion (*n* = 4), fibrin sheath formation (*n* = 3), malposition of the catheter tips (*n* = 4), central venous perforation (*n* = 1), thrombus formation (*n* = 12), regular catheter exchange without determined lesions (*n* = 3). Interventional catheter salvage procedures were performed, such as catheter exchange, balloon disruption of a fibrin sheath, angioplasty for central vein stenosis, and stent deployment. The technical success rate for catheter insertions was 100%, and no procedure-related severe complications were observed. The 30-day catheter patency for all assessable catheters was 85.2%.

**Conclusion:**

The use of MSCTV showed abnormal findings in almost 88.9% of cases concerning dysfunctional TCC. In this study, the examined appropriate endovascular techniques were found to be safe and technically successful, with a low incidence of procedure-related complications.

## Introduction

Functional vascular access is of crucial importance for patients undergoing hemodialysis (HD) (1). Although arteriovenous access is widely acknowledged as preferred option for most HD patients (2, 3), tunneled cuffed catheters (TCCs) remain a vital means of vascular access for a significant number of patients with end-stage renal disease under certain circumstances, such as dialysis requirements of >2–3 weeks, as a “bridge” to renal transplantation or maturation of peritoneal or arteriovenous access, in patients with preexisting failed or complicated arteriovenous access, and in elderly, diabetic and hypertensive patients who have difficulties in arteriovenous access creation and maturation (1, 2). The prevalence of TCCs usage varies from 2% to 49%, depending on geographical region (4). However, the use of catheters carries the highest risk of adverse events, such as infection, mechanical kinking, malpositioning of the catheter tip, thrombus accumulation, growth of a fibrin sheath, and inadequate blood flow, leading to poor dialysis adequacy (4–11). Various medical management protocols have been developed to address these problems, such as flushing the TCC lines with fibrinolysis protocols, reversing the TCC lines, and even TCC exchange (2, 12, 13). However, repeated attempts, which are sometimes performed inappropriately, increase the risk of complications, such as TCC damage or a catheter-related infection (13–15). Therefore, quickly and effectively determining the cause of TCCs dysfunction and guiding clinical treatments is essential for patients with a history of TCCs. In this study, we reported a series of cases in which MSCTV was utilized to evaluate TCC dysfunction and to provide guidance for clinical therapy.

## Material and methods

### Patients

This retrospective review of the interventional radiology database included patients who underwent radiologically salvage TCCs placement between July 1, 2016 and January 31, 2021. This study enrolled a total of 27 patients (19 female, 8 male) who underwent salvage procedures. The mean age of the patients was 62.89 years (range 32–89 years). Causes of renal failure among the patients were glomerulonephritis (*n* = 2), systemic lupus erythematosus with renal involvement (*n* = 2), diabetes mellitus (*n* = 5), hypertensive nephropathy (*n* = 6), renal calculus (*n* = 4), and undetermined causes (*n* = 8). The indications for TCCs placement included advanced age (*n* = 8), failed renal transplantation (*n* = 2), and failed graft or fistula access (*n* = 17). All patients had a history of prior TCCs insertion, and the reasons for the in-hospital TCC replacement were as follows: catheter dysfunction (*n* = 15), long-term duration and regular catheter exchange (*n* = 6), symptomatic extremity and/or facial edema (*n* = 5), catheter extrusion (*n* = 1). TCC dysfunction was defined as an inability to maintain adequate extracorporeal blood flow without prolonging the prescribed HD treatment, inability to flush the locking solution from TCC lines, blood flow rate <300 ml/min, arterial pressure <250 mmHg, high venous pressure >250 mmHg, or the need for TCC lines reversal (2, 16, 17). All the lesions were confirmed through MSCTV. The study procedure was conducted in accordance with institutional guidelines, and written informed consent was provided by all patients. All patients were regularly followed up at the nephrology/dialysis clinic in our institution.

### MSCTV images

Prior to the endovascular procedure, MSCTV scanning was conducted on each patient using a 256-slice scanner (Aquilion One, Toshiba Co, Tokyo, Japan) from the mandibular plane to the diaphragm plane. To obtain the volume dataset, 80–100 ml non-ionic contrast material (Ultravist 300, 300 mg I/ml, Bayer-Scheming, Leverkusen, Germany) was injected via the contralateral limb using a power injector at the rate of 4 ml per second, followed by normal saline injection. The technique of intelligent trigger was used for MSCTV scanning with the following parameters: 120 kV, 300 mA, 0.5 s, 0.625 mm × 64 mm, and 0.6–0.75 mm reconstruction thickness with an interval of 0.4 mm. Then MSCTV analyses were conducted using three-dimensional workstation software (Vitrea Enterprise Suit Workstation, Vital Images, Inc., Minnesota, USA) by two experienced radiologists. HD was arranged immediately after MSCTV examination and endovascular therapies for remove the contrast agents as soon as possible.

### Details of endoluminal procedures

Patient preparation involved the assessment and confirmation of essential hematologic parameters, including platelet count (≥50,000/L), hemoglobin level (≥8 g/dl), and International Normalized Ratio (≤1.5). A pair of fellowship-trained interventionists subsequently performed the endoluminal procedures under sterile conditions and local anesthesia (2% lidocaine). Patients were continuously monitored with electrocardiography and pulse oximetry.

Improper catheter tip placement can be salvaged using a guidewire if the catheter has adhered to contralateral veins. If a tunneled catheter is extruded, catheter exchange is indicated *in situ*. Fibrin sheath disruption may be appropriate if identified through MSCTV. In cases where the tip of the TCC is confirmed to be in the mediastinum through MSCTV and venography, a covered stent can be used to cover the tear. Symptomatic central thrombosis requires removal of the TCC and consideration of endovascular treatments.

### Follow-up

The technical success of the new catheter was defined as having at least one successful session of HD. Patients were followed up to 30 days after TCC exchange.

## Results

**Figure 1:**
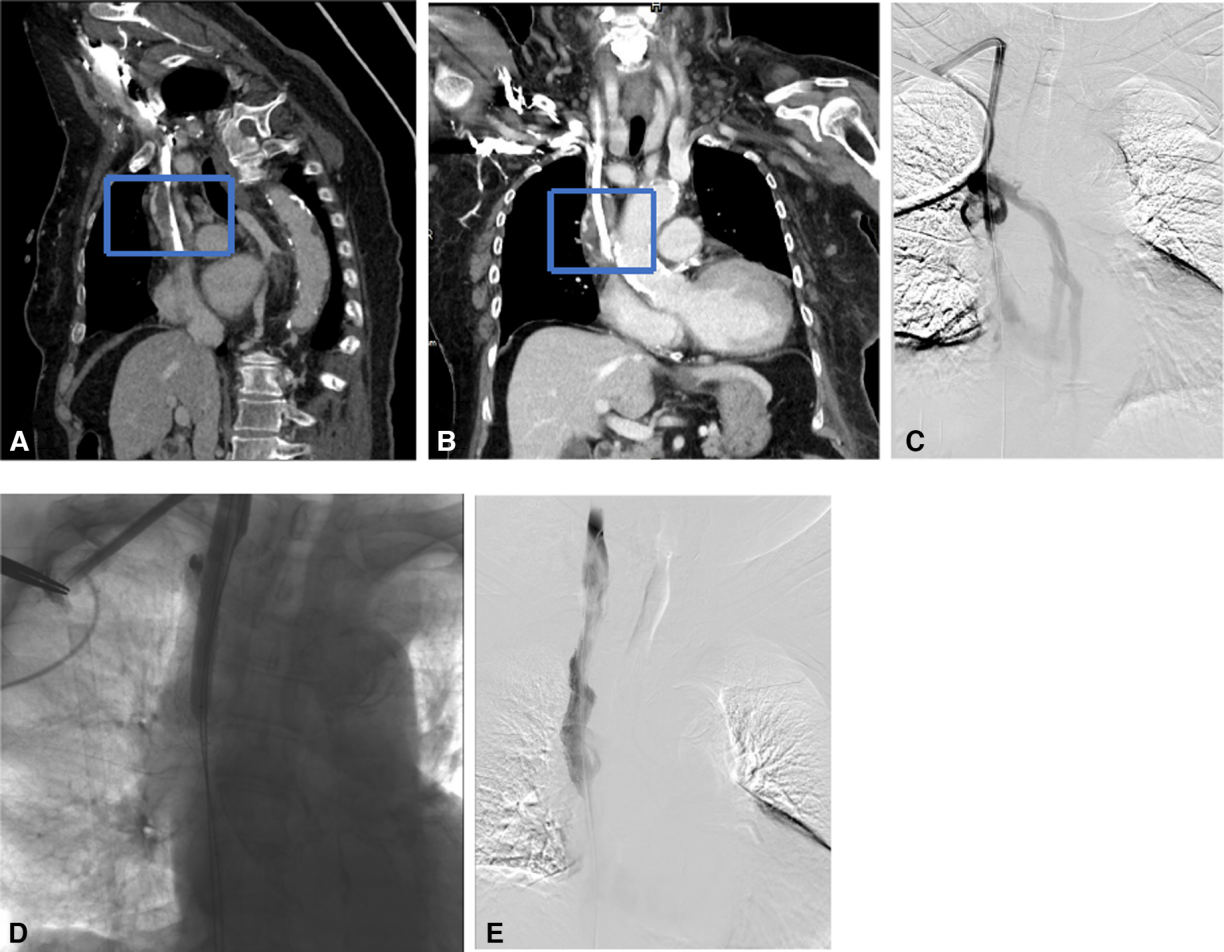
Recanalization with a bare stent for superior vena cava occlusion due to massive thrombosis. (**A**,**B**) CTV in the coronal and sagittal plane showed that the superior vena cava was occluded by massive thrombosis (the blue dotted box) at the catheter tip. (**C**) Venogram shows SVC occlusion with dilated azygos vein. (**D**) Recanalization was performed using a 14 mm × 80 mm balloon and a 14 mm × 80 mm bare stent. (**E**) Repeated angiography showed complete restoration of the blood flow.

**Figure 2:**
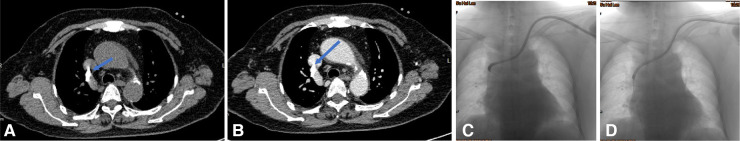
Endoluminal treatment for the catheter malposition into the azygos vein. (**A**,**B**) Transverse CT plain and enhancement scan showed the catheter malposition into the azygos vein. (**C**,**D**) Using a guidewire under fluoroscopic control, the catheter was partially withdrawn to the correct position.

**Figure 3:**
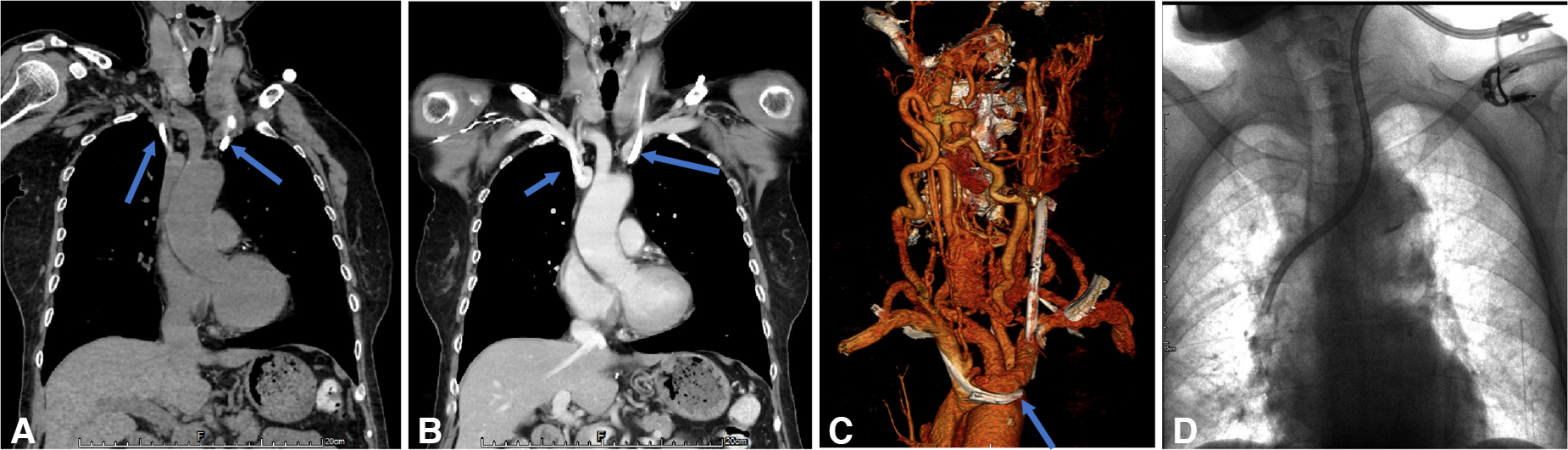
Endoluminal treatment for the catheter malposition into the contralateral innominate vein. (**A–C**) CT plain and enhancement scan showed the catheter malposition into the right innominate vein from the left side. (**D**) Exchanging the catheter under fluoroscopy with a guidewire is performed.

**Figure 4:**
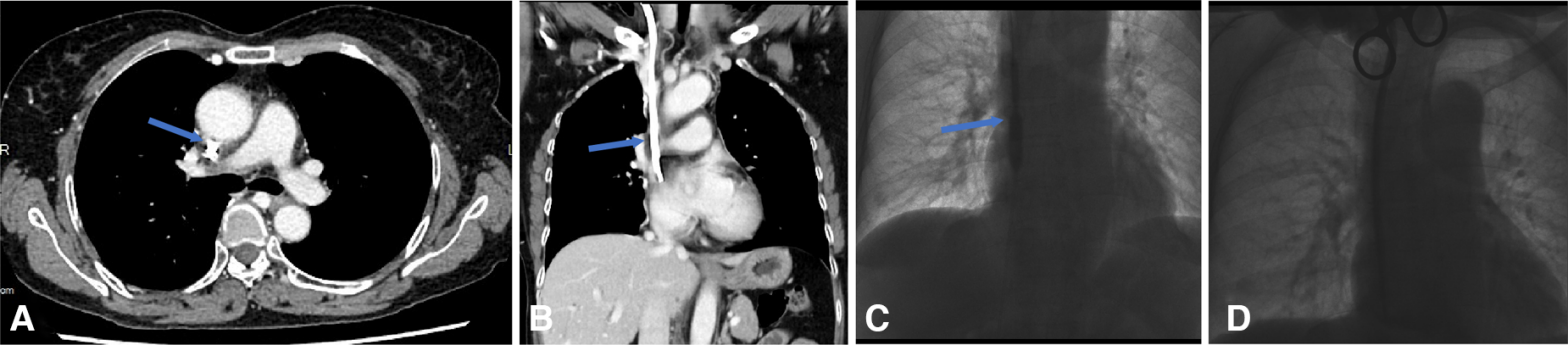
Endoluminal Dilation for the fibrin sheath. (**A**,**B**) CTV reveals fibrin sheath formation in the axial plane and coronal plane, as the underlying cause of the port malfunction. (**C**,**D**) The digital subtracted image: (**C**) a 10 mm × 60 mm balloon is inserted over a guidewire into the venous lumen of an embedded tunneled dialysis catheter and inflated along the length of the catheter from its distal tip to the cuff. The balloon and the CVC are then removed followed by inserting a new tunneled dialysis (**D**) over the guidewires and through the initial subcutaneous tract.

**Figure 5:**
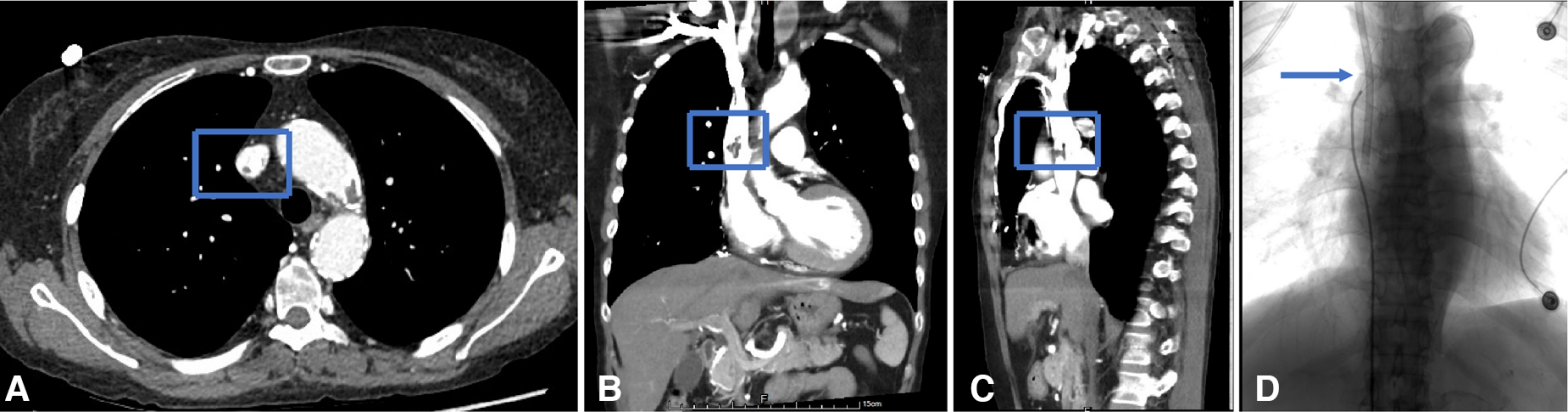
Endoluminal treatment for the fibrin sheath obstructing the catheter. (**A–C**) CTV reveals fibrin sheath formation in the axial, coronal, and sagittal planes. (**D**) When the catheter was withdrawn, the catheter tip appeared stuck to the right lateral sidewall of SVC. The fibrin sheath was stripped with a loop snare created by a 0.18-in guidewire, after which the malfunctional CVC was removed, and a new hemodialysis catheter was advanced.

**Figure 6:**
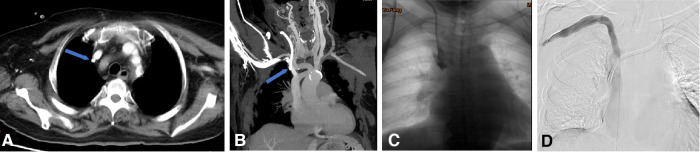
Endoluminal treatment for central venous perforation during dialysis catheter insertion. (**A,B**) The computed tomography thorax's transverse plane and coronal reformat showed the perforation site outside the right innominate vein (blue arrow). There was no attendant hemo/pneumothorax that otherwise required immediate intervention. (**C**) The central venogram showed contrast outlining in the mediastinum, confirming the CT findings of catheter perforation of the right innominate vein. (**D**) A covered stent was subsequently used to cover the rupture when the catheter was pulled into the internal jugular vein. Next, the right subclavian venography showed blood flow smoothly in the stent without contrast extravasation. Then, a new cuff CVC was inserted from the internal jugular vein into the superior vena cava.

The demographic characteristics of cases are shown in Table 1. Even though all patients had received anticoagulant treatment and thrombolytic therapy with urokinase, they were unable to resume normal catheter function. MSCTV was successfully utilized in all patients to assess the catheter's shape, tip position, the existence of a thrombus around the catheter or patency problems with the central vein. Out of 27 patients, 4 patients had central venous occlusion (2 in the SVC and 2 in brachiocephalic vein) (Figure 1). Catheter tips or peripheral thrombosis was observed in 12 patients, malpositioning of catheter tips occurred in 4 patients (2 patients with malpositioning in the azygos vein and 2 patients in the contralateral central veins) (Figures 2, 3). Fibrin sheath formation was identified in 3 patients (Figures 4, 5). Additionally, regular catheter exchange occurred without determined lesions in 3 patients, and 1 patient had central venous perforation (Figure 6).

**Table 1:** Patients’ characteristics (data are given as number, percentage, range or mean  ±  standard deviation).

Patients (*n*)	27
Sex: female/male	19/8
Age (range, median)	62.89 ± 15.91 (32–89)
Duration of line/dwell times (months, range)	23.61 (1 day–10 years)
Etiology of renal failure	
Glomerulonephritis	2
Diabetes mellitus	5
Renal calculus	4
Hypertensive nephropathy	6
Systemic lupus erythematosus	2
Unknown	8
Reasons for TCCs	
Advanced age	8
Failed renal transplantation	2
Arteriovenous access dysfunction	17
Reasons for TCC removal/replacement	
Catheter dysfunction	15
Symptomatic extremity and/or facial edema	5
Catheter extrusion	1
Long-term duration	6

Appropriate interventions were given to the patients based on the MSCTV results. In patients with central vein occlusion or massive thrombosis, the catheters were removed. After performing PTA and/or deploying covered stents, a new TCC was inserted. Directly catheters exchange was conducted for cases with a small amount of thrombosis. Endovascular repositioning was performed for 2 patients with malpositioned catheters in the azygos vein, 1 case in central collateral veins, while 1 patient with catheter malpositioned for a long time in central collateral veins was exchanged with a new catheter. Disruption of fibrin sheath was done for the catheters with fibrin sheath formation, and new catheters then replaced the removed ones. Catheters identified with no abnormalities after undergoing MSCTV screening were extracted, as slight intraluminal thrombosis was found near the tips, causing incomplete occlusion. Accordingly, catheter exchange was performed. Anticoagulant therapy was administered to all patients for at least 3 months, and no severe complications were noted during the procedure.

The 30-day catheter patency rate during follow-up was 85.2%. Nonetheless, during the follow-up period, two catheters were extracted due to suspected catheter-related infection and catheter tip malposition.

## Discussion

Given their considerable advantages, which include relative stable hemodynamics, immediate use after insertion, pain-free procedure and relatively easy insertion, tunneled cuffed catheters (TCCs) are becoming a preferred option among hemodialysis (HD) patients when no other vascular accesses is available, or when arterio-venous fistula (AVF) is impossible or not suitable for patients with limited life expectancy (2, 18). Consequently, the population of patients receiving TCC as the final HD option requires significant care and attention to sustain its patency (2, 19, 20). The present study showed that, compared to other well-established causes of catheter dysfunctions, such as central venous stenosis, fibrin sheath formation, and thrombus formation, the occurrence of mechanical problems, such as catheter malposition, was relatively low, which is consistent with previous studies (21). Dysfunction immediately noted after TCC placement is usually caused by mechanical problems, such as catheter malposition or venous perforation, which, albeit rare, can lead to an emergency resulting in high mortality rates (11, 22). However, gentle equipment handling and careful fluoroscopic monitoring of the guidewire, dilator, and peel-away sheath placement through Digital Subtraction Angiography (DSA) can avoid this catastrophic event (23). Dysfunction occurring after successful initial use of TCCs is often caused by various factors such as catheter migration, central venous occlusion, central venous thrombosis, and fibrin sheath formation, as reported in the present study. Hence, it is essential to promptly recognize imaging appearances and initiate appropriate immediate action to optimize the outcomes of dysfunctional TCCs.

Chest x-ray is recommended as a routine examination initially to exclude catheter malpositioning or kinking (24). However, it is not enough to evaluate the lumen of catheters and the central veins thoroughly. When x-ray and ultrasound fail to detect abnormalities, DSA is recommended to assess the catheters and the central veins thoroughly (25). However, it is costly and requires a high dose of contrast material, making it more suitable when therapeutic intervention is inevitable. Vascular Doppler sonography is a widely used non-invasive imaging test, both for guiding TCC placement and postoperative assessment. Nonetheless, it has limitations, as images could be confused with those of bones and lungs (26, 27). Due to the limitations of ultrasound in the thoracic cavity, culprit lesion can typically be detected and verified by CT venography, which is noninvasive and enables visualizing all three extremities simultaneously (2, 28). MSCTV has also shown to be effective in assessing catheter-associated venous diseases (29). MSCTV is effectively used in our center as a problem-solving tool in complex cases where conventional imaging techniques have failed to provide sufficient data, including catheter shape, the position of the tip, the presence of thrombus around the catheter, and the patency of central veins.

In HD patients, urgent intervention is necessary in case of TCC dysfunction to prolong the primary vascular access. Surgical intervention can cause significant surgical trauma, and conservative medical treatment fails to effectively solve the problem, especially if fibrin sheath and massive thrombosis occurred. Fibrin sheath is a sleeve of fibrin and proteinaceous material that surrounds the catheter, which affects 40%–100% of TCCs (2). The stuck catheter is always diagnosed at removal time, often leaving operators unprepared. Forceful removal of these catheters can cause severe substernal pain, avulsion of the vena cava, catheter breakage, and potentially fatal vascular lesions. Many techniques described in the literature can be used to treat fibrin sleeves, such as mechanical disruption or replacement and replacing a malfunctioning catheter (30). In our center, we prefer to perform angioplasty/stripping of the fibrin sheath followed by catheter exchange, a method that is also recommended by the KDOQI workgroup (2). Fibrin sheath stripping was found to be less effective than over-the-wire catheter exchange for long-term patency, so this approach was not recommended (31). Catheter survival was reported to be significantly improved by utilization of the sheath disruption technique compared to catheter exchange alone (32). Although the literature on these techniques has shown mixed efficacy, we recommend starting with the least invasive intervention, transcatheter lytic therapy, followed by catheter exchange with balloon disruption of the fibrin sheath or fibrin sheath stripping. However, it is important to avoid excessive pulling to prevent catheter disconnection from the port or catheter fracture. In the present study, we achieved a 100% success rate using this method. Disruption the intravascular fibrin sheath is vital to prevent immediate repeat occlusion by the same fibrin sheath. We achieved this by advanceing the catheter at least 1 cm deeper than its original position. Intraluminal thrombosis is identified as the primary cause of TCC dysfunction (33). While thrombolytic therapy has an excellent initial success rate of over 80%, its 2-month patency can be as low as approximately 36% (34). Endovascular treatments are often the primary treatment option with favorable results (35, 36). The first-line treatment for thrombus-related occlusion in our center is endovascular treatment, which refers to either PTA alone or PTA combined with percutaneous transluminal stenting (35–40). In case of minor catheter tip thrombosis, the catheter can be directly replaced based on anticoagulation. For patients with massive thrombosis and/or venous occlusion, the procedures were carried out as described previously (37). A 0.035-inch stiff guidewire was inserted through the dysfunctional TCCs, and the flossing wire technique was used to recanalize the lesion. Then, PTA and an appropriate covered stent was deployed. Venous injuries occur less frequently than 1% (41), however, appropriate and prompt actions are crucial in the initial management of this potentially fatal complication (11, 42). This complication is partly due to the large caliber of TCCs, which can lead to severe hemorrhage. Although open surgical repair was a conventional treatment for venous perforations (43, 44), endovascular stent repair is an attractive and minimally invasive alternatives (45, 46). In the treatment process, it is important to note that the catheter should only be removed after deploying the covered stent to control the perforation, as demonstrated in our case. Malpositioning is a possible complication that can occur after initial insertion. Repositioning the catheter with a guidewire and exchanging the catheter under fluoroscopy are effective solutions. According to a retrospective study, this meathod had a success rate of 65% at the bedside and 100% under fluoroscopy, which is consistent with our results (47). This problem is particularly noteworthy when there is a prior history of central venous catheter insertion.

Following are some suggestions for the placement of TCCs: firstly, the use of fluoroscopy for placing catheters is strongly recommended, especially for patients with a history of catheterizations. DSA-guided catheterization can monitor the position of the catheter tips in real-time and reduce the chance of vascular injuries. Secondly, when facing the dysfunction of the catheter, if CT is not available, an x-ray examination should be carried out to exclude the malposition of the catheter tip before using urokinase. Thirdly, for patients with multiple catheterizations, fluroscopy should be used to monitor the insertion in real-time or catheter exchange, simultaneously treating vascular diseases. Higher success rates and lower complication rates have been reported for radiologically TCC placement. The 100% technical success rate reported in the present retrospective series further validated the radiologic data for image-guided catheter placement.

There are some limitations in the present study, including the small sample size. Further clinical control study in which MSCTV is being directly compared with DSA is underway to identify the specificity and sensitivity of MSCTV.

## Conclusion

MSCTV provides accurate and sufficient information for assessing dysfunctional TCCs. This non-invasive imaging technique provides detailed, high-definition, and three-dimensional reconstruction of the TCC and surrounding structures. Additionally, investigation of patient history helps with planning for the rescue of the catheter and in some instances to help with replacement of the catheter. Interventional placement of TCCs under DSA is a safe and technically successful procedure.

## Data Availability

The original contributions presented in the study are included in the article/Supplementary Material, further inquiries can be directed to the corresponding author.
